# Retraction: Long non‐coding RNA LINC00607 silencing exerts antioncogenic effects on thyroid cancer through the CASP9 promoter methylation

**DOI:** 10.1111/jcmm.18251

**Published:** 2024-03-20

Lanzhen Li, Zhongcheng Gao, Lei Zhao, Peiyou Ren, Hongyan Shen. Long non‐coding RNA LINC00607 silencing exerts antioncogenic effects on thyroid cancer through the CASP9 Promoter methylation. J Cell Mol Med. 2021; 25: 7608–7620. https://doi.org/10.1111/jcmm.16265.

The above article, published online on 07 July 2021 in Wiley Online Library (wileyonlinelibrary.com), has been retracted by agreement between the authors, the journal Editor‐in‐Chief, Stefan Constantinescu, The Foundation for Cellular and Molecular Medicine and John Wiley and Sons Ltd. The authors asked to retract the article as they found that the ARO cell line used in the study has been contaminated with a HT‐29 derivative, and therefore the conclusions of the paper are not supported. Subsequently, an investigation into concerns raised by a third party, revealed inappropriate duplication of images between this and other articles that were previously published in a different scientific context. Thus, the editors consider the conclusions of this manuscript substantially compromised. Although the authors initially asked for the retraction, when asked to approve the retraction wording, they remained unresponsive.

